# Splice and Dice: Intronic microRNAs, Splicing and Cancer

**DOI:** 10.3390/biomedicines9091268

**Published:** 2021-09-19

**Authors:** Alex C. H. Wong, John E. J. Rasko

**Affiliations:** 1 Gene & Stem Cell Therapy Program, Centenary Institute, University of Sydney, Camperdown 2050, Australia; a.wong@centenary.org.au; 2 Sydney Medical School, University of Sydney, Sydney 2006, Australia; 3 Cell and Molecular Therapies, Royal Prince Alfred Hospital, Camperdown 2050, Australia

**Keywords:** microRNA, introns, cancer, alternative splicing, mirtrons, splicing factors, DROSHA

## Abstract

Introns span only a quarter of the human genome, yet they host around 60% of all known microRNAs. Emerging evidence indicates the adaptive advantage of microRNAs residing within introns is attributed to their complex co-regulation with transcription and alternative splicing of their host genes. Intronic microRNAs are often co-expressed with their host genes, thereby providing functional synergism or antagonism that is exploited or decoupled in cancer. Additionally, intronic microRNA biogenesis and the alternative splicing of host transcript are co-regulated and intertwined. The importance of intronic microRNAs is under-recognized in relation to the pathogenesis of cancer.

## 1. Introduction

MicroRNAs (miRNAs) are short single-stranded non-coding RNA of approximately 22 nucleotides (nt) in length. First discovered in 1993 in *C. elegans* [1], miRNAs provide an evolutionally conserved mechanism by which gene downregulation is mediated [2]. MiRNAs, together with the Argonaute proteins, form the RNA-induced silencing complex (RISC) through which miRNAs target messenger RNA (mRNA) via base-pair complementarity [3]. Targets with high base-pair complementarity induce mRNA transcript degradation and a strong silencing effect, whereas targets with partial complementarity promote translational repression and a milder effect [4,5]. In this way, a single miRNA can exert widespread effects by downregulating multiple functionally-related genes [6]. MiRNAs provide a powerful modulation of biological pathways affecting cancer development, progression, and treatment resistance via oncogenic or tumor suppressor roles [7,8,9].

Introns are segments of genetic code that are typically removed during or after transcription of RNA from DNA in a process termed splicing. As they were observed to be generally removed from mature messenger RNA (mRNA), introns were originally regarded as “junk” genetic material [10]. Advances in high-throughput sequencing and bioinformatics have revealed introns as evolutionarily conserved regulators of gene expression via multiple mechanisms [11,12,13]. Alternative splicing of introns occurs in an estimated 92% of human genes, allowing each gene to transcribe multiple combinations of exons which greatly expand proteomic diversity [14]. Intron retention, whereby introns are preserved in mature mRNA, adds an additional layer of gene regulation via post-transcriptional downregulation either through nuclear detention or cytoplasmic nonsense-mediated decay of mRNA [15,16]. Introns can also enhance gene expression by modulating the rate of gene transcription, and regulate the nuclear export and stability of mRNA transcripts [13].

Introns also harbor miRNA as well as other non-protein-coding genomic elements including long non-coding RNAs, small nucleolar RNAs, small interfering RNAs and Piwi-interacting RNAs [17]. Although miRNAs can also be located across exons or splice junctions, intronic miRNAs are the most common form of intragenic miRNAs, representing around 60% of all known miRNAs [18]. Given that introns only span around 25% of the genome [19], the predilection of miRNAs to reside within introns is worthy of further study. In contrast to intergenic miRNAs that are transcribed via independent promoters, intronic miRNAs are often transcribed together with their host genes using the same promoters; in this situation, both are derived from the same molecule of pre-mRNA [20,21,22]. Co-expression of intronic miRNAs and their host genes allow an additional layer of functional complexity whereby intronic miRNAs can either synergize or antagonize host gene functions [23,24]. Moreover, intronic miRNA processing can cooperate or compete with splicing of its host gene in ways that are yet to be fully characterized [25]. In this review, we explore the current knowledge of the biogenesis of intronic miRNAs, their regulation with respect to interactions with intron splicing, and their implications to cancer biology.

## 2. Biogenesis of Intronic miRNAs

**Figure 1:**
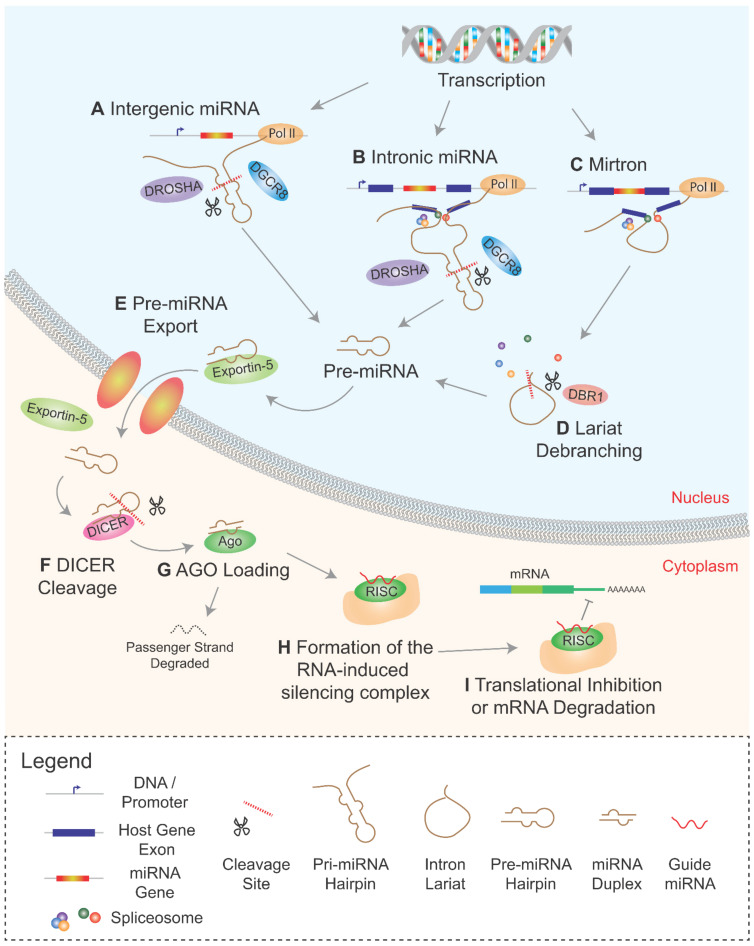
Overview of miRNA Biogenesis. (**A**) Intergenic primary miRNAs (pri-miRNAs) undergo co-transcriptional microprocessing via DGCR8/DROSHA to produce precursor miRNA (pre-miRNA). (**B**) In intronic miRNAs, microprocessing occurs during intron commitment for splicing and generally completes before the intron is spliced. (**C**) Mirtrons are a special class of intronic miRNAs whereby pre-miRNAs are derived directly from intron splicing and (**D**) lariat debranching by DBR1. (**E**) Canonical and mirtron pre-miRNAs join a common pathway of Exportin-5 mediated cytoplasmic export, after which (**F**) DICER cleavage produces a miRNA duplex. (**G**) miRNA duplexes are loaded onto Argonaute (Ago) proteins whereby strand selection occurs. (**H**) Retention of the miRNA guide strand by Ago is critical to the formation of the RNA induced silencing complex (RISC) which (**I**) targets protein-coding mRNAs promoting either transcript degradation or inhibiting translation. Pol II = RNA Polymerase II.

Most miRNAs are generated via the canonical miRNA pathway (Figure 1A) [26]. The initial substrate is the primary miRNA (pri-miRNA), a linear molecule of RNA directly transcribed from DNA. Pri-miRNA transcription is most often mediated by RNA polymerase II, although some are transcribed via RNA polymerase III [27]. Characteristically, pri-miRNAs contain regions of self-complementarity that form hairpin secondary structures critical for downstream processing [28]. Two stretches of self-complementary RNA base-pair, often imperfectly, to form the hairpin stem, with the intervening conserved sequence of bases forming the hairpin loop [29]. This hairpin recruits the microprocessor complex which consists of DiGeorge Syndrome Critical Region 8 (DGCR8) and the ribonuclease III DROSHA [30]. The hairpin stem of a canonical pri-miRNA is typically around 33 nt in length, of which DROSHA cleaves to remove the 11 nt of the lower stem, producing precursor miRNA (pre-miRNA) [30].

DROSHA cleavage via its ribonuclease III activity results typically in pre-miRNA with a 2 nt 3′-overhang at its base, which is in turn recognized by Exportin 5 (Figure 1E) [31]. Binding by Exportin 5 facilitates the cytoplasmic export of pre-miRNA [32]. Following cytoplasmic export, the pre-miRNA hairpin loop recruits DICER, which cleaves the loop thereby producing a miRNA duplex (Figure 1F) [33]. This duplex is then loaded onto Argonaute proteins whereby strand selection takes place (Figure 1G) [34]. The “passenger” strand is unwound and ejected from the Argonaute complex and is subsequently degraded. The retained “guide” strand programs the Argonaute to hone in on transcripts of its target genes, leading to formation of the RISC (Figure 1H) [34]. The RISC binds miRNA target sites which are typically situated at 3′-untranslated regions (UTRs) of protein-coding transcripts but can also reside on 5′-UTRs and protein coding regions (Figure 1I) [35]. RISC binding can either act to promote mRNA degradation or inhibit mRNA translation [4,5].

### 2.1. Co-Transcriptional Processing of Intronic miRNAs

Intragenic miRNAs, including intronic miRNAs, are expressed at levels that correlate with their host genes [20]. This substantiate the idea that, although an estimated 35–50% of intronic miRNAs can also be transcribed via independent promoters [27,36], the majority were derived from the same strand of RNA as that of the spliced host gene transcripts [20]. Intron splicing results in the formation of a circular molecule of RNA with a short tail, known as an intron lariat. Initially, it was thought that intron splicing and subsequent debranching of the intron lariat by Debranching RNA Lariats 1 (DBR1) was required before its resident miRNAs underwent DROSHA processing [37]. Later studies showed that microprocessing of pri-miRNA was predominantly co-transcriptional, and intronic miRNAs were microprocessed concurrently with intron splicing [22]. DGCR8/DROSHA binds to and cleaves the pri-miRNA hairpin directly from the intron loop whilst the flanking exons and the intervening intron are tethered to the committed spliceosome (Figure 1B) [21,22]. Microprocessing of intronic miRNA thereby results in a disconnected intron which is subsequently degraded by exonucleases that facilitate the completion of splicing [38]. Thus, the interconnectivity between microprocessing and intron splicing implies an overarching complex mechanism that co-regulates both pre-miRNA biogenesis and splicing.

### 2.2. Mirtrons and the Non-Canonical miRNA Pathway

Around 500 human intronic miRNAs, termed mirtrons, bypass DROSHA-dependent microprocessing [39]. Instead, mirtron pre-miRNAs are derived directly from intron splicing and intron lariat debranching (Figure 1C/D) [39,40]. Mirtrons contain sequences that self-complement to form the ~22 nt hairpin stems analogous to DGCR8/DROSHA mediated cleavage products of canonical miRNA. This contrasts with the 33- nt hairpin of typical canonical DROSHA-dependent miRNAs [41]. Debranched mirtrons are analogous to canonical pre-miRNAs and join the canonical pathway at the cytoplasmic export stage by Exportin-5 (Figure 1E). Mirtrons are subsequently processed by DICER in the same manner as canonical miRNAs [41].

Mirtrons, first identified in invertebrates, were observed to contain the precise 2- nt 3′-AG overhang created by their splice acceptor motifs, identical to pre-miRNA derived from DROSHA microprocessing [41]. In contrast, mammalian mirtrons did not strictly have these 2-nt overhangs, but instead have both 5′-G and 3′-G overhangs [42]. Such mirtrons are also exported by the canonical pathway since Exportin-5 accepts a broader range of hairpin substrates than the classical 3′-AG overhangs [42,43]. Additionally, mirtron pre-miRNAs are not strictly bound by their intron boundaries. Mirtrons can be “tailed” by extra nucleotides at their 5′- or 3′-ends (or both), which are subsequently cleaved by nuclear exonucleases prior to pre-miRNA export [44]. In 3′-tailed mirtrons, this occurs via the RNA exosome [45], in which the mirtron is protected from exonucleolytic degradation due to the secondary structure of their hairpins [44,46]. 5′-tailed mirtrons are presumably trimmed via another yet-to-be characterized mechanism prior to nuclear export. 5′-tailed mirtrons are the most abundant type of mirtrons (with 420 annotated), followed by canonical mirtrons (33 annotated) and 3′-tailed mirtrons (18 annotated) [39].

### 2.3. Comparisons and Contrasts between Mirtrons and Canonical Intronic miRNAs

Mirtrons represent the largest class of non-canonical miRNAs. They are structurally distinct from canonical miRNAs, although the differences are more complex and do not solely depend on a single parameter [47]. A recent machine-learning approach showed that, compared to canonical miRNAs, mirtrons have a 5′-arm richer in guanine and thereby 3′-arm richer in cytosine [47]. Also, mirtrons have higher free energy levels and shorter hairpin lengths [47].

The DROSHA-independent, splicing-dependent nature of mirtron biogenesis affords several advantages compared with canonical miRNAs. First, mirtrons are not affected by saturation of the microprocessor. DROSHA is a multi-functional protein and is involved also in mRNA destabilization, transcriptional regulation, and maintaining genome integrity [48]. Impairment of DROSHA microprocessor activity, as occurs in mutant tumor protein p53, is implicated in tumorigenesis [49]. Additionally, amplification of oncogenic pri-miRNAs saturates the microprocessor, leading to indirect downregulation of other DROSHA-dependent miRNAs [50]. Secondly, mirtrons evolve more quickly than canonical miRNAs [2]. This may in part be attributed to the fact that their expression only requires one cleavage event (DICER) instead of two [51]. Thus mirtrons require shorter hairpins and consequently a shorter stretch of self-complementary sequences than conventional miRNAs [51]. From an evolutionary perspective, mirtrons are younger and less conserved across species. For example, Wen and colleagues identified 478 and 488 mirtrons in human and mouse, respectively. Of these, only 13 mirtrons were conserved [39].

Mirtrons are less abundant than canonical miRNAs and the vast majority of mirtrons have yet to be functionally characterized. However, a small number of mirtrons play important oncogenic or tumor suppressor roles in multiple cancers. A list of the most well-characterized mirtrons and their roles in cancer are summarized in Table 1.

## 3. Interplay between miRNA Processing and the Spliceosome

The enrichment of miRNA residence in introns and the dependence of the microprocessor complex on transcription [22] suggest that intronic miRNA processing and intron splicing are co-regulated processes. Splicing is performed by the spliceosome, a dynamic mega-dalton complex of five ribonucleoproteins and hundreds of other auxiliary proteins [154]. The spliceosome forms as splicing factors are recruited stepwise onto nascent RNA initiated by splice site recognition [155]. Splicing progresses as the spliceosome undergoes the necessary conformational changes to ligate exons and excise the intervening intron [154]. RNA-bound splicing factors modulate splice site choice by enhancing or hindering splice site recognition [156]. Emerging evidence indicates that the microprocessor interacts with the spliceosome [38,157], substantiating the idea that splicing factors not only modulate splice site choice and alternative splicing, but also the expression of intronic and other intragenic miRNAs. In this section, we explore the role of the spliceosome and alternative splicing on regulating miRNA biogenesis.

### 3.1. Kinetic Regulation of Microprocessing and Splicing

The rate of RNA transcription is known to influence the outcome of alternative splicing, whereby a slower transcription rate favors the inclusion of alternate exons, and a fast rate promotes exon skipping [158]. Likewise, transcriptional stalling also facilitates miRNA biogenesis. Minigene studies have shown that increased nascent RNA retention at transcription sites induces higher miRNA expression [159]. Transcriptional stalling can be induced by exons flanking intronic miRNAs, or by removal of its polyadenylation signal [159]. Consistent with this, miRNA expression is enhanced by spliceostatin-mediated splicing inhibition [157].

**Figure 2:**
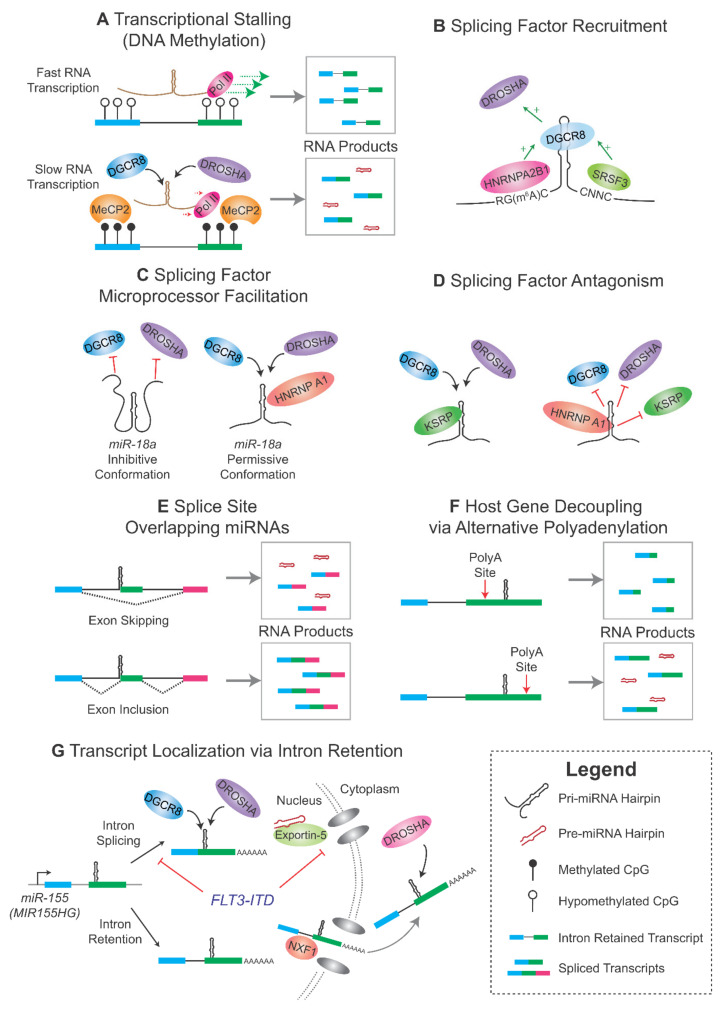
Examples of Splicing Regulation of Intronic and Intragenic miRNAs. (**A**) Transcriptional stalling (as occurs due to DNA hypermethylation) promotes microprocessing of intronic miRNAs. (**B**) Splicing factor binding adjacent to miRNA hairpins promotes recruitment of DGCR8/DROSHA. (**C**) Splicing factor binding to miRNA hairpins can alter pri-miRNA accessibility to microprocessor binding. (**D**) Splicing factors compete for binding to miRNA hairpins to facilitate or hinder pri-miRNA microprocessing. (**E**) Inclusion of alternate exons inhibits the expression of splice site overlapping miRNAs. (**F**) Alternative polyadenylation decouples miRNA/host gene expression. (**G**) Intron retention modulates pri-miRNA localization. Refer to the text (Section 3) for details. Pol II = RNA Polymerase II; PolyA = polyadenylation.

More recently it has been shown that increased DNA methylation near miRNA hairpins facilitate microprocessing by inducing transcriptional stalling (Figure 2A) [160]. Methylated CpG residues bind Methyl-CpG Binding Protein 2 (MeCP2) leading to stalling of RNA polymerase II, which in turn enhances microprocessing [160]. MeCP2 is known to interact with DGCR8 and may facilitate recognition of miRNA hairpins [160,161]. Interestingly, we and others have shown that DNA methylation also regulates intron retention and other forms of alternative splicing [162,163]. Higher methylation levels at alternate exons promote their inclusion [162], whereas lower levels of methylation in flanking exons promote intron retention [163]. In intron retention, decreased methylation leading to loss of MeCP2 binding impairs the recruitment of the splicing factor Transformer 2 Beta Homolog (TRA2B) [163]. Taken together, DNA methylation modulates both alternative splicing and miRNA processing via MeCP2 binding, which induces both transcriptional stalling and modulating recruitment of co-factors.

However, transcriptional stalling may not adequately account for the observed changes in miRNA expression. Instead, transcriptional stalling facilitates the context-dependent recruitment of microprocessor components, in coordination or competition with recruitment of other RNA binding factors that modulate splicing [164]. Supporting this, the presence of flanking splice sites does not always facilitate microprocessing. For example, *miR-211* microprocessing was dependent only on the presence of its upstream 5′-splice site, whereas *miR-204* microprocessing was dependent on both 5′- and 3′-splice sites [165]. The context dependent modulation of miRNA processing by auxiliary splicing factors is addressed in the next section.

### 3.2. Splicing Factors Regulating Microprocessing

RNA-bound splicing factors modulate splice site choice by enhancing or hindering splice site recognition. Many classes of splicing factors exist, and two major classes are well characterized. The serine-arginine (SR) proteins generally promote splice site recognition and exon inclusion, while heterogenous nuclear ribonucleoproteins (hnRNPs) generally promote exon skipping [156]. Additional complexities can arise when, for example, splicing factors regulate alternative splicing of other splicing factor genes [166]. This section discusses the role that splicing factors play in modulating the microprocessing of specific intronic miRNA.

Of the SR proteins, Serine and Arginine Rich Splicing Factor 1 (SRSF1) was the first splicing factor that was shown to also regulate miRNA processing. Wu et al. identified 4 miRNAs (*miR-7*, *miR-29b*, *miR-221*, and *miR-222*) which exhibits increased expression following SRSF1 overexpression [167]. SRSF1 regulates the microprocessing of *miR-7*, mediated by an SRSF1 binding motif on its lower stem [167]. Moreover, miR-7 directly targets the 3′-UTR of *SRSF1*, forming a negative feedback loop to regulate SRSF1 levels [167]. More recently, SRSF1 regulation of *miR-222* was shown to occur via a different mechanism [168]. The *miR-222* locus overlaps a splice site of a 44-nt mini-exon of its host gene, *MIR222HG*. SRSF1 binding to the mini-exon increases exon inclusion thereby precluding the production of *miR-222*. Blocking the SRSF1 binding site to the mini-exon increases *miR-222* expression, whereas blocking the SRSF1 binding site in the *miR-222* stem loop increases spliced *MIR222HG* expression [168]. Taken together, SRSF1 binding sites can either enhance or impair microprocessing depending on the context of their binding sites.

Mirtrons are formed directly from intron splicing and thus their biogenesis should be expected to be regulated by splicing factors. Consistent with this, *SRSF1* overexpression significant increases *miR-1229-3p* expression, whereas SRSF2 overexpression significantly increases *miR-1227-3p* and *miR-1229-3p* expression [169]. The largest effects were associated with splice enhancer motifs for SRSF1 and SRSF2 in their corresponding proximal exons, suggesting splice enhancer SR proteins may play a direct role in regulating mirtron expression through enhancing splice site recognition [169].

SRSF3 also enhances the microprocessing of a subset of miRNAs (Figure 2B). SRSF3 binds a CNNC motif adjacent to the basal junction of the pri-miRNA stem [170]. SRSF3 binding to this motif enhances the recruitment of DROSHA in a spatially-dependent context, whereby DROSHA cleavage in miRNAs with CNNC motifs were only enhanced when the motif was located ~17 nt from the DROSHA cleavage site [170]. In this manner, SRSF3 regulates a specific subset of miRNAs, including *miR-16*, *miR-30a* and *miR-142* [170,171].

Various hnRNPs have been implicated in modulating miRNA processing. The first such hnRNP to be identified was the splicing factor hnRNP A1. This factor binds to, and facilitates, the specific processing of *miR-18a* [172], which is the second member of the *miR-17-92* cluster of miRNAs. The *miR-17-92* cluster is a polycistronic locus of 6 miRNA residing mostly on the third intron of its host gene *MIR17HG* (formerly known as *C13orf25*). It is the first locus of polycistronic miRNAs to be classified as oncogenic and was the first classified “oncomiR” [173], being first identified as a region of amplification in B-cell lymphoma [174]. These miRNAs regulate important processes including cell cycle, proliferation and apoptosis, and are overexpressed in lymphoma, leukemia and diverse solid cancers [175]. hnRNP A1 facilitates *miR-18a* microprocessing by binding to the stem-loop sequence of *miR-18a*, which leads to the partial unwinding of its hairpin, thereby facilitating access to the microprocessor complex (Figure 2C) [176]. Knockdown of *hnRNP A1* selectively impairs the expression of *miR-18a* without changing the expression of the other five miRNA in the *miR-17-92* cluster [172], demonstrating the specificity of this layer of microprocessor regulation.

The KH-type splicing regulatory protein (KSRP) primarily degrades mRNA by binding to AU-rich elements in their 3′-UTR [177], but it can also facilitate exon inclusion by binding to nearby intronic splice enhancers [178]. Interestingly, KSRP also enhances miRNA processing by binding GGG-triplets in miRNA stem-loop sequences [179]. Additionally, this interaction may be competitive with that of hnRNP A1. In the case of miRNA *let-7a*, hnRNP A1 binding displaced that of KSRP, thereby inhibiting the microprocessing of *let-7a* (Figure 2D) [180].

### 3.3. Role of RNA m^6^A Modification

The N6-methyladenine modification of RNA (m^6^A), where the amine at the 6th carbon position of adenine is methylated, is the most common RNA epigenetic modification. Recent studies have shown that m^6^A is required for the recruitment of DROSHA and other splicing factors that modulate microprocessing [181,182]. Co-transcriptionally, RNA polymerase II (Pol II) directly recruits DGCR8 to the nascent transcript, which in turn recruits DROSHA. This process depends on Cyclin Dependent Kinase 9 mediated phosphorylation of the C-terminal domain of Pol II [183]. DGCR8 recruitment to miRNA hairpins is facilitated by m^6^A modification of GGAC residues in pri-miRNAs [181]. Consistent with this, knockdown of the gene encoding the m^6^A writer Methyltransferase-like 3 led to reduction in the expression of many miRNAs [181].

Two hnRNPs, HNRNPA2B1 and HNRNPC, regulate microprocessing by binding to motifs that involve m^6^A modifications. HNRNPA2B1 binds the RNA methylation mark m^6^A in RGAC motifs at the basal junction of pri-miRNA hairpins [182]. This binding facilitates the recruitment of DGCR8 which in turn recruits DROSHA (Figure 2B) [182]. HNRNPC, known to bind to m^6^A residues in GRACH motifs [184], was shown to facilitate *miR-21* processing [185]. *miR-21* expression was dependent specifically on HNRNPC binding to its pri-miRNAs upstream of the hairpin. HNRNPC-regulated *miR-21* expression targets the Programmed Cell Death 4 gene, thereby facilitating metastasis in glioblastoma [185]. Taken together, co-transcriptional RNA methylation facilitates microprocessing via the recruitment of DGCR8/DROSHA, HNRNPA2B1 and HNRNPC.

### 3.4. The Microprocessor as a Splicing Factor

Increasing evidence indicates that the microprocessor can regulate alternative splicing. Its splicing role was first characterized in the Eukaryotic Translation Initiation Factor 4H gene where DROSHA promotes skipping of its fifth exon [186], since this alternative exon forms a DROSHA substrate hairpin. Although DROSHA could cleave this exon as a microprocessor product, the effect of DROSHA on exon skipping is independent of its cleavage function, as dominant negative cleavage deficient DROSHA could also promote exon skipping [186].

In fact, *DROSHA* itself is a substrate of DROSHA-mediated exon skipping [187]. The 5′-splice site demarcating the seventh exon from its downstream intron forms a DROSHA substrate hairpin. DROSHA binding promotes the skipping of this exon [187]. Interestingly, skipping of exon 7 promotes nuclear localization of DROSHA whereas exon inclusion promotes cytoplasmic localization [187,188]. Although canonical miRNA processing occurs in the nucleus, emerging evidence indicates DROSHA can also process miRNAs in the cytoplasm [189]. Alternative splicing of *DROSHA* can also downregulate its activity by generating a unstable truncated DROSHA isoform (exon 27a inclusion) or a cleavage-impaired isoform (exon 32a inclusion) [190]. The enhanced expression of these novel DROSHA isoforms in various cancer datasets suggest that alternative splicing is actively exploited to downregulate microprocessing in cancer [190].

### 3.5. Alternative Splicing and miRNA Biogenesis

Alternative splicing and processing of intronic and other intragenic miRNAs can interact in myriad cooperative or competitive mechanisms. Splice site overlapping miRNAs (SO-miRNAs) exemplify the competitive relationship that miRNA processing can have with alternative splicing [191,192]. The inclusion of the alternate exon across which SO-miRNAs overlap results in removal of the intronic portion of its pri-miRNA sequence, thereby inhibiting its processing (Figure 2E). Conversely, microprocessing of SO-miRNAs lead to removal of the splice site, thereby inhibiting exon inclusion. We discussed earlier the role of SRSF1 in regulating the exon inclusion harboring SO-miRNA *miR-222*, thereby inhibiting *miR-222* expression [168]. Another example is the tumor suppressor *miR-34b* which resides on the acceptor splice site of the sole intron of its host gene. Minigene studies showed that intron splicing competitively inhibits the generation of *miR-34b* [191].

Melamed et al. detailed several characteristics peculiar to SO-miRNAs [192]. Firstly, SO-miRNA expression is typically lower than that of exonic or intronic miRNAs. This is exemplified by the example of murine *miR-412*, which resides on a 3′-splice site of its host gene *Mirg*. *Mirg* also contains 9 additional miRNAs residing either on introns or exons. *miR-412* was expressed at substantially lower levels than its neighboring intronic *miR-541* or exonic *miR-410* [192]. Moreover, *miR-412* expression was inversely proportional to the strength of its overlapping splice site [192], suggesting splice site recognition is in competition with microprocessor binding to SO-miRNA hairpins. Supporting this, DROSHA expression was inversely proportional to inclusion of the exon harboring the SO-miRNA *miR-412* [192]. Moreover, *miR-412* expression was inversely proportional to the inclusion ratio of its overlapping exon [192]. This allowed *miR-412* to be differentially expressed in different tissues, in contrast to the other intronic or exonic miRNAs on the same host gene [192].

The competitive relationship between splicing and microprocessing may be a context-dependent phenomenon. For example, the DROSHA microprocessing of SO-miRNA had no general effect on exon inclusion levels in host genes [193]. To explain this discrepancy with earlier reports, the authors of this study proposed that after SO-miRNA processing, the remaining transcript often does not produce a spliced product, but instead leads to premature transcript termination [193].

Alternative splicing can also uncouple the expression of intronic miRNAs from their host genes (Figure 2F). For example, the Myosin Heavy Chain 7B *(MYH7B)* gene harbors *miR-499* on its 19th intron. Skipping of the 7th exon in *MYH7B* causes a frameshift and introduces a premature termination codon in its 9th exon. In this way, exon skipping destines the transcript for cytoplasmic nonsense-mediated decay and consequent downregulation of *MYH7B*. This occurs without affecting *miR-499* expression levels [194]. Intragenic miRNAs can also be decoupled from host gene expression via alternative polyadenylation. For example, *miR-21*, residing on the terminal exon of its host gene Vacuole Membrane Protein 1, can be downregulated by the alternative use of a polyadenylation site proximal to the *miR-21* locus [195].

Alternative splicing can also affect miRNA expression by altering the cellular localization of its host transcript (Figure 2G). A recently characterized example is mediated by the FMS-like tyrosine kinase 3-internal tandem duplication (*FLT3-ITD*) mutation in acute myeloid leukemia (AML), where the *FLT3-ITD* upregulates *miR-155* expression via intron retention in its host gene [189]. *FLT3-ITD* is a well-characterized poor-prognostic mutation in AML [196], and *miR-155* is a well-established oncomiR that also confers poor prognosis [197]. *FLT3-ITD* up-regulates *miR-155* in AML via two coordinated mechanisms. Firstly, *FLT3-ITD* mediated SPRED1 phosphorylation led to the inhibition of Exportin-5, broadly blocking the export of canonical pre-miRNAs [189]. Secondly, *FLT3-ITD* increased phosphorylation of the splicing factor DDX3X, thereby impairing the intron splicing of the host gene of *miR-155*. These intron-retaining transcripts were exported to the cytoplasm via an alternate pathway mediated by Nuclear RNA Export Factor 1 (NXF1), bypassing the Exportin-5 blockade. Subsequently, *miR-155* pre-miRNAs were microprocessed by cytoplasmic DROSHA isoforms [189]. This complex mechanism selectively enhances the expression of oncogenic *miR-155* relative to the expression of other miRNAs.

Taken together, alternative splicing can affect intragenic miRNA processing in myriad ways, depending on their transcriptomic contexts.

## 4. Intronic miRNAs—Host Gene Interactions and Their Roles in Cancer

The most important functional effect of miRNAs residing within introns is the consequent coupling of miRNA expression with its host gene, allowing for either synergistic or antagonistic interactions within the miRNA-host gene pair [24]. Most host genes are expressed in a positively correlated relationship with that of their intronic miRNAs [24,198]. Gene ontology analysis of miRNA-host gene pairs showed that intronic miRNAs target biological processes more significantly related to the function of their host genes than could be expected by chance [24]. This is supported by the observation that intronic miRNAs with functional association with their host genes are more likely to be evolutionarily conserved [199]. A balanced number of intronic miRNAs have a synergistic or antagonistic relationship with their host genes [24]. Moreover, about 20% of all intronic miRNAs are predicted to directly target their host genes [200]. By targeting host genes, intronic miRNA can reduce the expression noise of their host gene, by quenching expression when there is a low level of host gene promoter activity [201].

Approximately 60% of all miRNAs reside in introns [18]. Introns are thought to be favorable regions for new miRNAs to arise, as intronic nucleotide repeats provide fertile ground for miRNA hairpins to evolve and these can be expressed without the need to co-evolve an independent promoter [202]. Consistent with this, older intronic miRNAs are more likely to be transcribed by an independent promoter [36]. Additionally, miRNAs in older host genes are more broadly expressed than those emerging in younger host genes [203]. França et al. explained these observations by separately considering miRNA emergence and fixation [203]. MiRNA emergence within introns are favorable as they co-opt host promoters, without the need to evolve their own promoter [203]. However, fixation of intronic miRNA depend on several factors. MiRNA fixation is favored if their host promoter provided a breadth of expression across tissues [203]. Alternatively, wider tissue expression could be induced by the evolution of an independent promoter [203]. Another factor is the functional utility that the miRNA-host gene interaction provides [199,203].

The predilection of miRNA to reside in introns demonstrates the utility of its interaction with its host gene, and this relationship can be either exploited or impaired in cancer. Numerous interactions between intronic miRNA and their host genes have been comprehensively reviewed elsewhere [23,198,204]. Here, we describe 3 examples of the more recently identified cancer-associated miRNA-host gene pairs in the following section. Other functionally characterized miRNA-host gene pairs are listed in Table 2.

### 4.1. mir-615-3p/HOXC5 Axis and Telomerase Regulation

*mir-615-3p*, residing in the intron of the Homeobox C5 (*HOXC5)* gene, is co-expressed along with its host gene but can also be expressed from its own promoter [213]. It primarily acts as a tumor suppressor gene but can also be oncogenic in some contexts [214]. For example, *mir-615-3p* inhibits tumor proliferation and metastasis in non-small cell lung cancer by targeting *IGF2* [215]. Conversely, *mir-615-3p* can promote proliferation and tumor cell migration and inhibit apoptosis in gastric cancer by targeting *CELF2* [216]. Importantly, *mir-615-3p* expression in differentiated cells target the *TERT* gene thereby leading to silencing of its gene product hTert, the protein subunit of telomeres. HOXC3 also downregulates *TERT* expression by interfering with the interaction between the *TERT* promoter and its enhancer [217]. Thus, the *mir-615-3p*/*HOXC5* axis represents a synergistic mechanism that regulates telomerase expression and thereby replicative potential in normal cells. Aberrant silencing of *HOXC5*, such as via *MUC1-C*/*BMI2* mediated silencing of HOX genes in breast cancer, can thereby disrupt this pathway and promote malignant transformation [218].

### 4.2. miR-374b/miR-545/FTX

Recently, a screen for co-expressed miRNA-host gene pairs in colon cancer identified *FTX* as an oncogenic host gene harboring several intronic miRNAs [18]. The two miRNAs residing in the first intron of *FTX*, *miR-374b* and *miR-545*, enhanced proliferation by targeting *PTEN* and *RIG-I*, respectively, thereby enhancing PI3K-Akt signaling [18]. Conversely, *miR-421*, residing in a downstream intron of *FTX*, had an anti-tumor effect by inhibiting the DROSHA-dependent processing of *miR-374b* and *miR-545* [18]. Additionally, *FTX* also interacts with DHX9 to promote aberrant adenosine-to-inosine RNA editing in multiple cancer cell types [18,219]. Thus, *FTX* overexpression along with its intronic miRNAs *miR-374b* and *miR-545* promote colon cancer via two different but synergistic oncogenic pathways.

### 4.3. miR-944/TP63 Axis and p53 Maintenance

The transcription factor p63, transcribed by the *Tumor Protein 63 (TP63)* gene, belongs to the p53 family of transcription factors along with p73 [220]. *miR-944* resides within the 6th intron of the *TP63* gene. p63 regulates cell type specification, differentiation, proliferation and apoptosis [220]. Alternate transcription start site usage in *TP63* gives rise to the truncated isoform ΔNP63α. This isoform is normally expressed in highly proliferating squamous cells but is also a potent oncogene in squamous cell cancer [221]. Upregulation of ΔNP63α is associated with cisplatin resistance, which occurs in part due to aberrant alternative splicing of apoptosis factors [222]. Additionally, ΔNP63α, in contrast to full-length p63, is a potent modulator of miRNA transcription and alters expression of multiple miRNAs including *miR-944* promoter-driven expression [223]. In colon cancer, *miR-944* stabilizes p53 by targeting the E3 ligases *COP1* and *MDM2* [224]. Thus, *miR-944* acts as a tumor suppressor intronic miRNA by antagonizing its host transcript ΔNP63α in squamous cell cancers.

## 5. Conclusions

Splicing and intronic miRNA biogenesis are intertwined processes and their co-regulation is currently underappreciated in cancer research. Introns are favorable locations for miRNA emergence and conservation through their ability to couple miRNA expression with functionally relevant host genes. Moreover, the myriad ways in which splicing factors can alter intronic miRNA expression adds yet another layer of complexity to gene expression regulation by miRNAs. These intricate networks of molecular relationships are either exploited or decoupled in cancer. Further characterization of intronic miRNAs with respect to alternative splicing and other RNA processing mechanisms is key to understanding how miRNAs are dysregulated in cancer and the development of new cancer therapies.

## Figures and Tables

**Table 1:** Top mirtrons in cancer.

Mirtron	Type	Host Gene and Intron	Validated Gene Targets	Cancer
*hsa-miR-877*	Mirtron	*ABCF1* Intron 12	*ACP5* [52], *AQP3* [53], *ATXN7L3* [54], *CCNA2* [55], *CD274* [56], *CDK14* [57], *CDKN2A* (upreg) [58], *FGF2* [59], *FOXM1* [60], *FOXP4* [61], *IGF1R* [62], *MACC1* [63], *MTDH* [64], *PIK3R3* [65], *PMEPA1* [66], *STARD13* [67], *SUZ12* [68], *TLR4* [69], *VEGFA* [70]	Bladder (TS) [58], Cervical (TS) [54,59,63], Colorectal (TS) [64], Gastric (TS) [53,56,70], Glioma (Onc) [69], Glioma (TS) [68], Laryngeal (TS) [61], Liver (TS) [57,60,65], Lung (TS) [52,55,62], Oesophageal (TS) [66], Pancreatic (Onc) [67]
*hsa-miR-1224*	Mirtron	*VWA5B2* Intron 17	*CREB1* [71], *ELF3* [72], *ETV1* [73], *FAK* [74], *KLF3* [75], *PGM5* [76], *PLK1* [77], *RSF1* [78], *SLC29A3* [79], *SP1* [80], *SND1* [81,82], *TGFBR2* [83], *TNS4* [84],	Breast (Onc) [76], Colorectal (TS) [80], Gastric (TS) [74,78], Glioma (TS) [71,83], Lung (TS) [73,75], Oesophageal (TS) [84], Osteosarcoma (TS) [77], Ovarian (TS) [81], Pancreatic (TS) [72], Prostate (TS) [82], Rectal (TS) [79]
*hsa-miR-1226*	Mirtron	*DHX30* Intron 20	*AKT1* [85], *AQP5* [86], *DUSP4* [87], *ERBB2* [85], *ITGB1* [88], *MUC1* [89], *PIK3R2* [85]	Breast (TS) [85,86,89], Liver (TS) [87,88],
*hsa-miR-1227*	Mirtron	*PLEKHJ1* Intron 1	*IRF2* [90], *MAPK13* [91], *SUPT16H* [92]	Endometrial (TS) [91], Lung (TS) [92], Osteosarcoma (Onc) [90],
*hsa-miR-1228*	Mirtron	*LRP1* Intron 48	*CSNK2A2* [93], *MIF* [94], *MMP14* [95], *SCAI* [96,97], *SOX17* [98], *TCF21* [99], *TP53* [100,101],	Breast (Onc) [96,98], Gastric (TS) [93,94,95], Liver (Onc) [101], Lung (Onc) [99], Osteosarcoma (Onc) [97], Ovarian (Onc) [100]
*hsa-miR-1229*	Mirtron	*MGAT4B* Intron 1	*APC* [102], *GSK3B* [102], *HIPK2* [103], *ICAT* [102], *ITGB8* [104], *MTOR* [105],	Breast (Onc) [102], Colorectal (Onc) [103], Glioma (TS) [104,105],
*hsa-* *miR-1236*	Mirtron	*RDBP* Intron 7	*AFP* [106], *ATG7* [107], *CHD4* [108], *CDKN1A* (upreg) [109], *HDAC3* [110], *HMGB1* [111], *HOXB7* [112,113], *KLF8* [114], *MTA2* [115], *SENP1* [110], *SLC9A1* [116], *TPT1* [117], *TRIM37* [118], *ZEB1* [119,120],	Breast (TS) [108,116,120], Cervical (TS) [118], Colorectal (TS) [112], Gastric (TS) [111,115], Glioma (TS) [113], Lung (TS) [107,114,117], Liver (TS) [106], Kidney (TS) [109], Ovarian (TS) [119],
*hsa-miR-937*	3′-tailed mirtron	*SCRIB* Intron 29	*APAF1* [121], *CCRL2* [122], *FOXQ1* [123], *INPP4B* [124], *SOX17* [125], *TIMP3* [126],	Breast (Onc) [121,125], Breast (TS) [123], Colorectal (Onc) [126], Lung (Onc) [124],
*hsa-miR-939*	5′-tailed mirtron	*CPSF1* Intron 4	*APC2* [127], *ARHGAP4* [128], *BCL2L1* [129,130], *CDH5* [131], *HDGF* [132], *IGF1R* [133], *JUNB* [134], *LIMK2* [135], *NGFR* [136], *SLC34A2* [137], *TIMP2* [138],	Breast (Onc) [131], Colorectal (TS) [130,135], Gastric (TS) [137], Lung (Onc) [138], Lymphoma (TS) [134], Ovarian (Onc) [127], Osteosarcoma (TS) [133], Pancreatic (Onc) [128], Prostate (TS) [132],
*hsa-miR-1292*	5′-tailed mirtron	*NOP56* Intron 11	*DEK* [139]	Gastric (TS) [139],
*hsa-miR-1976*	5′-tailed mirtron	*RPS6KA1* Intron 6	*PIK3CG* [140], *PLCE1* [141],	Breast (TS) [140], Lung (TS) [141],
*hsa-miR-4728*	5′-tailed mirtron	*ERBB2* Intron 25	*CAV1* [142], *COL1A2* [142], *EBP1* [143], *ESR1* [144], *MST4* [145], *PAPD5* [146], *THBS2* [142]	Breast (Onc) [143,146], Breast (TS) [145], Colorectal (TS) [142], Lung (TS) [147],
*hsa-miR-6838*	5′-tailed mirtron	*POLM* Intron 10	*GPRIN3* [148], *WNT3A* [149],	Breast (TS) [149], Gastric (TS) [148]
*hsa-miR-6852*	5′-tailed mirtron	*TLN1* Intron 24	*FOXJ1* [150], *LEF1* [151], *ICAM1* [152], *TCF7* [153],	Colorectal (TS) [153], Gastric (TS) [150], Glioma (TS) [151], Liver (TS) [152]

Top cancer-relevant mirtrons, with mirtron type and host intron location as annotated by Wen et al. [39]. Target genes are downregulated by their respective mirtrons; except those denoted “upreg” to indicate that the target gene is upregulated by non-canonical mechanisms. “TS” and “Onc” denote tumor suppressor and oncogenic roles, respectively, in their corresponding tumor types.

**Table 2:** Functionally associated miRNA/Host gene pairs in cancer.

miRNA	Host Gene	Cancer	Functional Association
*hsa-* *miR-196b*	*HOXA9*	Mixed lineage leukemia	Complex: miR-196b directly targets its host gene HOXA9 (oncogene) but also targets FAS (tumor suppressor) [205]
*hsa-* *miR-204*	*TRPM3*	Renal (Clear cell)	Antagonism: inhibits TRPM3-induced proliferation by directly targeting TRPM3; also inhibits oncogenic autophagy by targeting LC3B [206]
*hsa-* *miR-326*	*ARBB1*	Medulloblastoma	Synergism: inhibits E2F1-associated pro-survival function by directly targeting E2F1 (miR-326) and E2F1 acetylation (ARBB1) [207]
*hsa-* *miR-342*	*EVL*	Lymphoid leukemia	Antagonism: miR-342/EVL balance determines myeloid (high miR-342) or lymphoid (high EVL) differentiation. miR-342 antagonises EVL-induced lymphoid proliferation [208]
*hsa-miR-675*	*H19*	Breast	Synergism: both promote cell migration, invasiveness and stemness [209]
*hsa-* *miR-1204/ 1205/ 1207*	*PVT1*	Various	Synergism: PVT1 sponges multiple miRNAs, thereby enhancing tumor proliferation induced by miR-1204, miR-1205 and miR-1207 [210]
*hsa-* *miR-3189*	*GDF15*	Various	Antagonism: mir-3189 promotes p53-independent apoptosis; GDF15 is implicated in metastasis [211]
*hsa-* *miR-4728*	*ERBB2*	Breast	Synergism: inhibits apoptosis thereby promoting therapy resistance to HER2(ERBB2) inhibitors [212]

## Data Availability

Not applicable.
